# The two faces of cognitive motivation in cognitive stress: The stress-reducing effect of positive affect and the stress-intensifying effect of motivation

**DOI:** 10.1016/j.cpnec.2026.100362

**Published:** 2026-07-10

**Authors:** Felix M. Schweitzer, Sören Enge, Julian Hellmann-Regen, Monika Fleischhauer

**Affiliations:** a Institute of Psychosocial Research for Health Promotion and Intervention (IHPI), Department of Psychology, Faculty of Natural Sciences, MSB Medical School Berlin, Berlin, 14197, Germany; b Section Clinical Neurobiology, Department of Psychiatry and Psychotherapy, Campus Benjamin Franklin, Berlin Institute of Health, Charité – Universitätsmedizin Berlin, Corporate Member of Freie Universität Berlin, Humboldt-Universität zu Berlin, Berlin, 12203, Germany

**Keywords:** Need for cognition, Epistemic curiosity, Intellect, Stress, Cortisol, Alpha-amylase, Task motivation/task enjoyment

## Abstract

Cognitive motivation reflects, among other aspects, the dispositional tendency to seek, engage in, and enjoy cognitively stimulating activities as well as intellectual novelty. Individuals with high cognitive motivation should show higher state motivation and a more positive affect in cognitive challenges but its conceptualization might also suggest lower stress in such activities. Since the interplay of these variables has to our knowledge not yet been examined using a purely cognitive stressor, participants in the present laboratory study (*N* = 201) were exposed for 20 minutes to a series of highly demanding reasoning tasks. Subjective stress during the experiment was measured five times using the Negative Affect subscale of the PANAS and a single visual analogue scale (VAS) item. Salivary alpha-amylase (sAA) and cortisol were assessed as physiological stress indicators. Structural equation modelling was used to test whether cognitive motivation is directly associated with stress, and whether there exists an indirect effect via motivation to work on the tasks and the enjoyment of the tasks. Subjective stress was positively associated with motivation and negatively with task enjoyment, the indirect effect being significant only in case of the latter. No significant indirect effects were found with sAA or cortisol as outcomes, although the pattern of effects for sAA closely resembled the one observed for subjective stress. Interestingly, our findings suggest a bifurcated effect of cognitive motivation on stress during cognitive challenges: while positive affect might work as a protection, the motivation to face the challenge in fact rather seems to enhance it.

## Highlights

• Cognitive motivation did not directly predict subjective or physiological stress responses.
• Task enjoyment was associated with lower and task motivation with higher subjective stress, suggesting opposing affective and motivational paths.
• Task enjoyment significantly mediated the effect of cognitive motivation on subjective stress.
• SAA showed no significant mediation but a similar effect pattern.
• Findings reveal nuanced effects of cognitive motivation on stress during cognitive challenges.

## 1 Introduction

Psychological constructs reflecting the motivation to invest resources in intellectual activity have been related to higher engagement in cognitive tasks, better mood, and less frustration and have also been linked to positive health outcomes [1]. However, the conceptualization of such constructs also raises the question whether they could act as potentially stress-reducing factors during highly challenging cognitive activity. Given the ubiquity of cognitively demanding situations in daily life, examining cognitive motivation (CM) in this context may not only deepen our understanding of CM but could also provide a basis for health-related interventions.

### 1.1 Cognitive motivation

Within the last decades, research has sought to integrate different conceptualizations of the general motivational disposition to search for, engage with, and invest effort in cognitively taxing activity [e.g., 2,3]. As elaborated in the framework of “investment traits” [4, p. 841], this disposition may manifest in several closely related traits capturing characteristics of engaged and effortful information processing. Among the most prominent of these often independently studied constructs are the need for cognition (NFC), epistemic curiosity (EC), and intellect. Whereas NFC reflects the tendency to engage in and enjoy challenging cognitive activity [3], epistemic curiosity (EC) [5] represents a “drive to know” [6, p. 187] and the desire to reduce knowledge gaps [5,6]. Finally, intellect [7] captures behavioral and motivational tendencies related to intellectual performance [8]. These constructs differ slightly in focus with, for example, EC more strongly reflecting the joy of learning and intellect contributing perseverance- and creativity-related aspects [7]. However, despite differences in emphasis, research has shown high intercorrelations and hence questionable discriminant validity among these constructs [9,10], suggesting that they may be understood as manifestations of the suggested broader underlying motivational tendency. Reflecting this disposition, relations with cognitive and motivational outcomes have been observed, including self-control [11], goal orientation [12], cognitive effort investment [13], persistence in cognitive challenges [12], exploratory behavior [5,9], and creativity [[14], [15], [16]]. In line with previous research [e.g., 17,18], we refer to this common disposition as “cognitive motivation” (CM). Rather than representing a single observable construct, CM can hence be assumed to manifest in the mentioned closely related NFC, EC, and intellect, capturing aspects of the willingness to seek out, engage in, and persist in cognitively challenging activity.

### 1.2 Cognitive motivation and stress during intellectual challenges

Cognitively demanding situations can be stimulating but also stressful [19]. According to the transactional stress model [20], individuals responding to stress first assess whether a situation is harmful, threatening or challenging and whether coping resources suffice. The subsequent coping can then be problem-focused – i.e., targeting the stressor – or emotion-focused – i.e., regulating emotional responses. Stress is proposed to arise when perceived demands exceed resources [20].

In cognitively demanding contexts, individuals high in CM may be more likely to appraise tasks as manageable or challenging rather than threatening. Since, for example, NFC relates positively to fluid intelligence to a small-to-moderate degree [21], such higher cognitive abilities and hence potentially reduced perceived task difficulty may facilitate this appraisal. Furthermore, their experience in deliberation may yield more accurate evaluations and prevent maladaptive reactions. But even when appraising a task as difficult, high-CM individuals may address the problem more constructively and hence cope more effectively [22].

Beyond the subjective experience of stress, physiological responses involve the activation of the hypothalamic-pituitary-adrenal (HPA) axis, releasing glucocorticoid cortisol, and the autonomic nervous system (ANS), releasing adrenaline and noradrenaline. Both enable adaptations to perceived threats and are essential for effective coping [23]. Cognitive appraisal processes have been suggested to not only affect subjectively experienced stress, but also physiological responses [20,24], with anticipatory appraisal explaining as much as 35% of variance in cortisol responses [25]. Prior research specifically on CM associations with HPA and ANS activation in response to stressors is currently sparse. However, negative associations of the CM-related openness/intellect with heart rate and cortisol [26] levels as well as blood pressure in response to experimentally induced stress have been reported [27]. While there are also findings to the contrary [28], an explanation for better physiological adaptation in high-openness/intellect may be the tendency to explore and thereby cope with stressors cognitively [27,29]. Since this propensity for cognitive investment is likely shared between openness/intellect and the above-characterized CM, this also suggests better adaptation to physiological stress in high-CM individuals. Yet, given the considerations above, high CM may also affect physiological responses specifically to stress in *cognitive* challenges. Due to their frequent engagement in such activity, such individuals should habituate to it over time, potentially resulting in a less pronounced response to such potential stressors.

### 1.3 Task motivation and task enjoyment

Task motivation [30] as a situational, activity-specific construct [31] has been conceptualized based on cognitive theories of motivation such as Self-Determination Theory (SDT [32]). As discussed by [33], it involves cognitions and feelings elicited when engaging with a specific task such as appraisals of competence, task value, and costs [34,35]. Task motivation reflects the “here and now” of motivation in a specific situation, in contrast to more stable motivational dispositions [36, p. 293]. Nevertheless, it has been argued to be also affected by these traits [34].

Task enjoyment represents a positive affective reaction to task engagement, involving, for instance, pleasure and fun [37,38], with strong relations to other constructs such as motivation and performance [32,39,40]. However, task enjoyment and motivation can be conceptually distinguished by different roles in cognitive activity: while the former has been proposed to function as an evaluator of the attainment of current goals, motivation influences the pursuit of goals directly [41].

Frameworks such as Trait Activation Theory (TAT) conceptualize traits as “latent potentials” for a certain behavior, cognitions, or the experience of emotions in response to trait-relevant cues [42, p. 201]. Given that motivation to engage with and enjoy cognitive stimulation are core aspects of CM, this suggests higher situational motivation and enjoyment when presented with a challenging cognitive task.

Whereas CM has been empirically associated with greater willingness to invest effort in complex tasks [13,43], its role as a predictor of task enjoyment in such contexts does not yet seem to have been directly examined. However, it relates to less frustration and mental discomfort in such situations [3] and individuals high in openness/intellect have shown enhanced positive affect during laboratory stress [27]. Given their conceptualization, task motivation and enjoyment may also be associated with responses to a laboratory stressor. The enjoyment of a cognitive challenge and the appraisal of demands as manageable may attenuate stress [20], consistent with strong empirical evidence suggesting a buffering effect of positive affect [44]. The effect of motivation is less clear. Intrinsic motivation is conceptually related to flow experience, and hence, for example, a deep sense of control and reduced rumination [45]. However, empirically, positive relations between the motivation to work on a test and stress have been observed [e.g., 46]. This may reflect greater personal relevance of the task in highly motivated individuals, which, given high task demands, could amplify stress responses. In sum, task enjoyment and motivation might show differential relations with cognitive stress, the former being negatively, and the latter positively associated. Since both are assumed to be positively related to CM, this also suggests indirect effects, operating in opposite directions [47].

### 1.4 The present study

NFC, EC, and intellect likely share a common motivational disposition for engagement in cognitive activity beyond construct-specific nuances such as the desire for acquiring knowledge [5] or additional creativity-related aspects [7]. As discussed, proposed mechanisms like the cognitive exploration of stressors or prior experience with cognitive challenges are primarily related to CM as this common disposition. To focus on this conceptual overlap and achieve a parsimonious representation, we adopt an integrative approach on cognitive investment traits [e.g., 18], treating NFC, EC, and intellect as first-order indicators of second-order CM. Using challenging cognitive tasks, the present study consequently investigates whether this trait CM directly predicts subjective and physiological stress in a laboratory setting. Moreover, it is tested whether there are indirect effects via situational task motivation (TM) and task-specific enjoyment (TE) as core situational manifestations in such contexts. We hypothesize.

H1 CM is negatively associated with subjective (H1a) / physiological (H1b) stress over the course of the laboratory session.

H2 CM is positively associated with TM.

H3 CM is positively associated with TE.

Although the direction has not been conclusively determined in case of TM, conceptual and empirical considerations suggest that TM and TE are related to cognitive stress. We finally hypothesize.

H4 There is an indirect effect of CM on subjective (H4a) / physiological stress (H4b) via TE.

H5 There is an indirect effect of CM on subjective (H5a) / physiological stress (H5b) via TM.

## 2 Methods

### 2.1 Participants

Following established laboratory stress protocols [48], exclusion criteria were: severe physical, neurological, or mental disorders, prescription-only medication, cortisone-containing drugs, neurological, endocrinological, or psychiatric treatment within the past 12 months, and heavy smoking (>10 cigarettes/day). Based on these, 19 participants (8.48%) were excluded from the data. Following careless responding screening, another four participants (1.79%) with clear signs of inattentiveness were excluded. The final sample consisted of 201 German-speaking participants (70.15% female, 29.85% male; *M*_age_ = 23.47, *SD*_*age*_ = 3.61, range = 18–35 years). Out of these, 89.05% were native speakers, 72.14% were enrolled in undergraduate studies of psychology, and 14.43% in graduate studies of clinical psychology and psychotherapy.

### 2.2 Procedure

This study is part of the PERCOG project (see also [49]) and was preregistered prior to data collection on the Open Science Framework (OSF): https://osf.io/yv2e3/overview. Supplementary materials such as data, analysis code, research materials, and additional results can be accessed at: https://osf.io/v7qdn/overview.

**Fig. 1:**
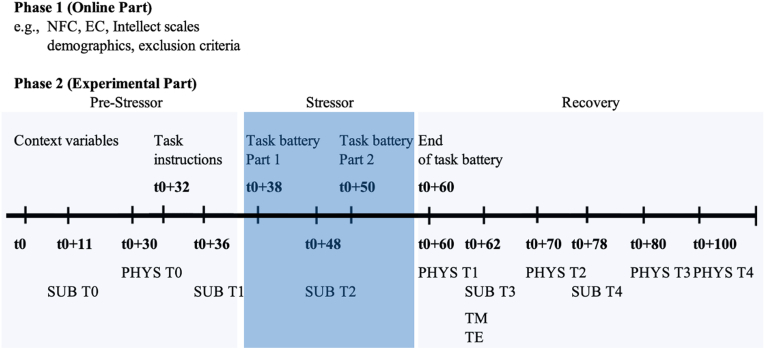
Procedure. NFC = need for cognition; EC = epistemic curiosity; SUB = subjective stress measurement; PHYS = physiological stress measurement; TM = task motivation; TE = task enjoyment.

Participants were recruited via the university's SONA online research application and the researchers' networks. In phase 1, participants completed a 40-minute online questionnaire via Tivian (v21.1–24.2) including personality assessments and screening for exclusion criteria. Participants were also instructed to refrain from alcohol (within 12 hours), caffeine and smoking (within 2 hours), as well as intense physical activity, eating, and tooth brushing (within 1 hour) before the subsequent laboratory session (phase 2), which on average took place 4.12 days later (*SD* = 4.81, range = 0–25). Upon arrival, participants confirmed adherence to pre-session instructions and were briefed on the study. The duration of the whole period of stress measurement was based on previous studies [e.g., 50], reporting an average acclimation time, post-stress until peak cortisol interval, and recovery time of 23, 13, and 46 minutes, respectively. Subjective markers were collected 11 (T0), 36 (T1), 48 (T2), 62 (T3), and 78 (T4) minutes after arrival, thus allowing to cover acclimation, pre-stressor levels, peak stress, and recovery. Timing of physiological markers was oriented on cortisol, which reflects arousal with a latency of 20-25 minutes [48]. To align them as closely as possible with subjective stress measurement, they were collected 30 (T0), 60 (T1), 70 (T2), 80 (T3), and 100 (T4) minutes after arrival (Fig. 1). After baseline measurement of physiological stress, participants received instructions and representative example items to familiarize them with the reasoning tasks. They then completed the task battery, followed by a recovery phase including further subjective and physiological stress measurements. The session ended after the final physiological stress measurement and a debriefing. This study was conducted in accordance with the Declaration of Helsinki (revised) and ethical approval was received by the Medical School Berlin Ethics Committee (MSB-2022/111).

### 2.3 Cognitive task battery

To elicit stress responses solely from cognitive challenges, we used a concise 20-minute battery of demanding validated intelligence tasks. This was divided into two 10-minute blocks, separated by a brief break for subjective stress measurement. The battery included matrix tasks from the Hagen Matrices Test [51], figural analogy and cube rotation tasks from the International Cognitive Ability Resource Project [52] (https://icar-project.com/, accessed on 25 October 2021), and anagram tasks [53].

The first three task types appeared 10 times in Block 1 and 9 times in Block 2 while anagram tasks appeared 20 and 18 times, respectively. Task order was fixed for all participants, starting with a matrix task, two anagrams, a spatial reasoning task, and a figural analogy task. Two consecutive anagrams were included to account for shorter processing times and ensure comparable time and effort across tasks. Task selection was based on item difficulties to ensure a significant cognitive challenge while broadly representing reasoning abilities, i.e. verbal, figural, and spatial reasoning. Mean item difficulty based on the original publications was .34 (*SD* = .21, range = .04–.86), ranging from .28 (*SD* = .09) for the cube rotation tasks to .36 (*SD* = .13) for the figural analogy tasks. The tasks were presented using SoSci-Survey.

### 2.4 Stress markers

Subjective stress was assessed with a visual analogue scale (VAS) ranging from 0 (completely disagree) to 100 (completely agree) presented on a computer and phrased: “The current situation is stressful for me”. Together with the VAS item, the 10-item Negative Affect (NA) subscale of the German Positive and Negative Affect Schedule (PANAS) [54] with a scale from 1 (very slightly or not at all) to 5 (extremely) was administered. Internal consistency ranged from ω = .85 to .89 across measurement times.

Salivary alpha-amylase (sAA) and cortisol (sCORT), reflecting activation of the sympathetic-adrenal-medullary (SAM) and HPA axis, respectively, were collected using synthetic cotton swabs and stored at −18°C. Biochemical analyses were conducted in the Laboratory for Neurobiology of the Department of Psychiatry, Charité - Universitätsmedizin Berlin. Samples were centrifuged (2 min at 1000 × g) and free cortisol was analyzed using a TR-FRET-based, adapted immunoassay (Cisbio International, Codolet, France). Time-resolved fluorescence was measured using a Clariostar multimode plate reader (BMG Labtech, Ortenberg, Germany) at 620 nm and 665 nm. Inter- and intraassay coefficients of variation (CV) were below 12%. A modified IFCC-based CNPG assay adapted for room temperature was used to determine sAA activity. Samples and standards were diluted 1:200, measured in triplicates, and absorbance was read at 405 nm at fixed intervals. Four-parameter nonlinear regression (*r*^2^ > .998) was used to quantify activity, with inter- and intraassay CVs below 10%.

### 2.5 CM, TM, and TE questionnaires

NFC was assessed using the 16-item German NFC scale [55] with a 7-point Likert scale (from −3 = completely disagree to 3 = completely agree). EC [56], focusing on the intrinsic pleasure of acquiring knowledge, was assessed with the German 10-item scale [57], using a 4-point Likert scale (from 1 = completely disagree to 4 = completely agree). Intellect was assessed with the 24-item scale [7] using a 7-point Likert scale (from 1 = completely disagree to 7 = completely agree). TM and TE were measured directly after the cognitive tasks using four self-designed items, one per task type. Example items on the matrix tasks were „How motivated were you to work on the matrix tasks?” and “How much did you enjoy working on the matrix tasks?“. They were answered on a 5-point Likert scale ranging from 1 (not at all) to 5 (very).

### 2.6 Data analysis

#### 2.6.1 Data preparation

Because stress markers were strongly right skewed across measurement occasions, Box-Cox transformations were applied [58]. Item parceling was used on the CM measures and parcels were formed based on loading patterns in tested item-level measurement models derived from the literature [59]. The area under the curve with respect to increase (AUCi) was determined [60], allowing to reduce data from repeated stress measurements into a single variable, offering flexibility in further statistical analyses. Positive AUCi values indicate that most of the curve lies above baseline (i.e., stress increase), while negative values reflect levels predominantly below baseline (i.e., stress reduction).

#### 2.6.2 Measurement and structural models

Structural equation modelling (SEM) was used to test hypotheses. The conceptual overlap and shared variance between NFC, EC, and intellect suggest that they represent different variations of a common underlying disposition towards cognitive activity, and CM was thus modelled as a second-order latent factor. Latent TM and TE were based on four task-specific items each. Since one item from each factor referred to the same task type (e.g., anagrams), within-task-type residuals were allowed to correlate to account for shared variance beyond the construct. Moreover, there is evidence linking task motivation with positive affect [32,61]. Although the focus of the present paper was not on that specific relationship, they may share variance beyond the prediction of CM and disturbances of TM and TE were hence allowed to correlate.

The two physiological AUCi markers shared very little variance. Instead of modelling a latent variable, each hypothesis (e.g., H1a) was tested in two separate models using manifest AUCi outcomes. A *conjunctive* approach to hypothesis testing [62] was chosen to address type I error accumulation, and a null hypothesis was thus considered rejected if the hypothesized effect was significant in *both* corresponding models (e.g., the model including sAA *and* the one including sCORT).

Prior research found age, sex, BMI, and hormonal cycle influencing the stress responses [[63], [64], [65]]. The main effects in the estimated models were hence controlled for age, BMI, and dummy-coded menstrual cycle phase in women (with men as reference) by modelling paths to the dependent variables. Groups in the latter were naturally cycling women in the follicular phase, in the luteal phase, using hormonal contraceptives, and irregular cycle (< 21 or > 35 days [e.g., 66]) or unknown cycle status.

To test H1a and H1b, a model including only the second-order CM factor, the AUCi as well as the covariates was specified. This was tested against a model in which the CM-stress AUCi path was fixed to zero. In a larger model, indirect paths via TE (H4) and TM (H5) were added to the model including CM and the AUCi. These were defined as the product of the paths from CM to TM and TE, respectively, and from the latter to the stress AUCi. The hypothesis of an indirect effect was rejected if the respective coefficient failed to reach statistical significance in at least one of the subjective (H4a and H5a) or physiological markers (H4b and H5b). To test whether alternative models better represented the data, three comparison models were tested. In the first, the direct path from CM to stress was fixed at zero, representing a full mediation model. In the other, either the path from TM or TE to the AUCi was fixed to zero, implying no indirect effect of CM on stress.

Traditional cut-off values for model fit are a CFI and TLI ≥.95, RMSEA <.06 and SRMR ≤.08 [67]. However, recent studies question the general applicability of these cut-offs [68]. We hence conducted simulations to additionally derive dynamic cut-off values for our models (Tables S7–8). Moreover, AIC and BIC were interpreted with lower values indicating better fit [67]. A robust maximum likelihood (MLR) estimator was used and the significance tests of indirect effects were based on 5000-sample bootstrapping.

After excluding careless responders, there remained cases with missing data due to unstable internet connections, experimenter errors, or low-quality saliva samples. Missing values were hence handled by estimating the models using full information maximum likelihood. The attainable sample was constrained by financial resources for participant recruitment and biochemical analyses. To evaluate whether the achieved sample size provided adequate power, post-hoc RMSEA-based power analyses were conducted to quantify the ability to detect and reject close fit under different levels of model misfit. Statistical analyses were conducted using R Studio, version 2024.4.2.764 [69]. See supplementary materials for details on all these procedures and software.

## 3 Results

### 3.1 Descriptive statistics and correlations

Descriptive statistics and intercorrelations of manifest and latent main variables can be found in the supplementary materials (Tables S2–6).

### 3.2 Measurement models

**Fig. 2:**
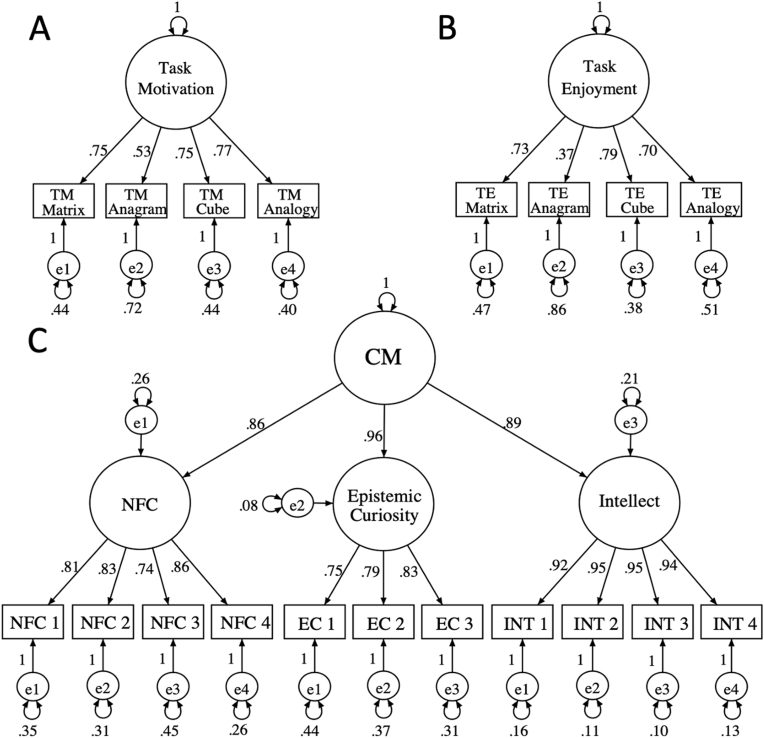
Measurement models for cognitive motivation, task enjoyment, and task motivation. CM = cognitive motivation; NFC = need for cognition; EC = epistemic curiosity; INT = intellect; TM = task motivation; TE = task enjoyment; A: TE measurement model; B: TM measurement model; C: CM measurement model.

Measurement models are displayed in Fig. 2 and model fits on these and the item-level models on which they were based can be found in the supplementary materials (Table S1). The EC measurement model was just-identified with three parcel indicators and the fit of the parcel indicator measurement model of NFC and intellect, second-order CM as well as that of TM and TE was good. Internal consistency was ω = .88 for NFC, ω = .97 for intellect, ω = .84 for EC, ω = .75 for TE and ω = .79 for TM.

### 3.3 Stress marker trajectories

**Fig. 3:**
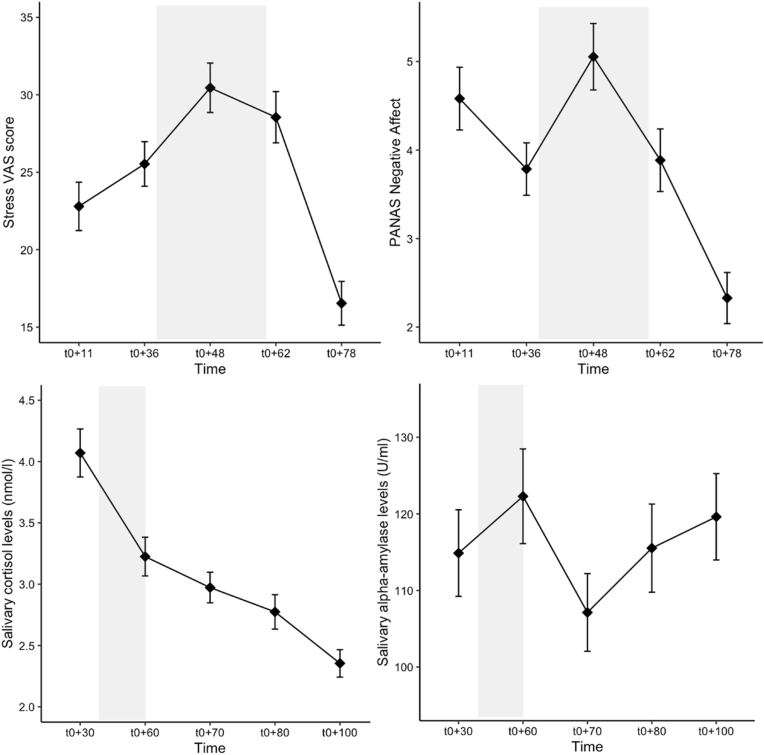
Individual stress markers during the laboratory session. PANAS = Positive And Negative Affect Scale; VAS = visual analogue scale; stressor period displayed in grey.

Fig. 3 displays the stress markers throughout the laboratory session. The specific trajectories of all participants on the four markers (Fig. S1) and results of one-sided paired Wilcoxon tests testing the significance of stress level increases are reported in the supplementary materials (Section 1.3, 1.4).

### 3.4 Structural equation models

#### 3.4.1 CM and stress

To examine H1a and H1b, we first estimated a SEM including only a path from CM to the AUCi, reflecting stress over the course of the session. In these models, neither the VAS stress item AUCi (β = .07, *p* = .327) nor the PANAS NA AUCi (β = .07, *p* = .315) were associated with CM. Regarding physiological stress, CM was also not significantly related to the sCORT AUCi (β = .02, *p* = .805) or the sAA AUCi (β = .06, *p* = .605). Hypotheses H1a and H1b are hence rejected.

#### 3.4.2 CM, TM and TE

Four distinct models additionally including TM and TE as mediating variables were estimated. The CM-TM path was significant (β = .20, *p* = .031) as was that to TE (β = .36, *p* < .001) in the subjective stress models. Effects were only marginally different (≤.01) in the physiological stress models. In sum, H2 and H3 are supported.

#### 3.4.3 TM and TE mediating the effect of CM on stress

**Fig. 4:**
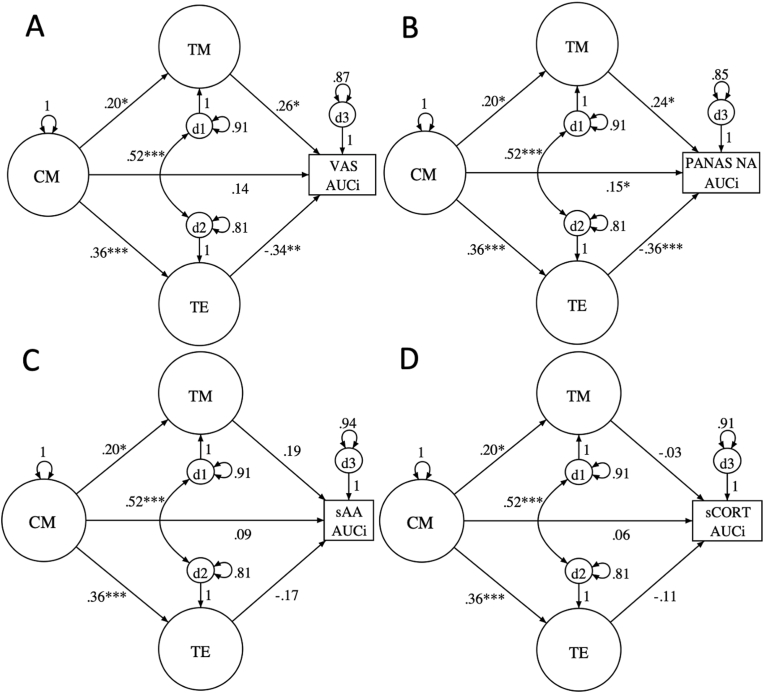
Structural models with different stress markers as outcome. CM = cognitive motivation; TM = task motivation; TE = task enjoyment; VAS = visual analogue scale stress item; PANAS NA = Positive And Negative Affect Scale subscale for negative affect; sAA = alpha-amylase; sCORT = salivary cortisol; AUCi = area under the curve with respect to increase; A: structural model with AUCi based on VAS item as stress outcome; B: structural model with AUCi based on PANAS NA as stress outcome; C: structural model with AUCi based on sAA as stress outcome; D: structural model with AUCi based on sCORT as stress outcome; covariate paths (age, BMI, dummy-coded hormonal status) and correlations between residuals of indicators of TE and TM that refer to the same task (e.g. anagram) not displayed for clarity; significance of residual variances not marked; *p* < .05*; *p* < .01**; *p* < .001***.

Table 1 reports the fit of the assumed partial mediation model together with models reflecting alternative representations of the data, for example implying no indirect effect via TM. Diagrams of the structural models are depicted in Fig. 4. Fit statistics suggest good fit of all hypothesized models. In the model including the VAS stress item AUCi, the effect of TM on the latter was β = .26 (*p* = .034), and the indirect effect was β = .04 (*p* = .160). The direct effect of TE on stress was β = −.34 (*p* = .002) and the indirect effect was β = −.12 (*p* = .014). The direct effect of CM on stress with both TM and TE included in the model was β = .14 (*p* = .071).

**Table 1:** Fit statistics for structural models on indirect effects of CM on stress AUCi via TM and TE.

Model	RMSEA [90% CI]	SRMR	CFI	TLI	χ^2^	*df*	*p*	Δχ^2^	Δ*df*	Δ*p*	AIC	BIC
VAS stress												
Partial mediation	.039 [.026, .051]	.053	.974	.968	341.18	260	.001				7321.31	7333.47
Full mediation	.040 [.026, .051]	.055	.973	.968	344.49	261	<.001	3.88	1	.049	7322.41	7334.43
No TE indirect effect	.041 [.029, .052]	.055	.970	.965	352.70	261	<.001	16.52	1	<.001	7330.66	7342.69
No TM indirect effect	.041 [.028, .052]	.055	.972	.966	349.07	261	<.001	8.49	1	.004	7327.29	7339.32
PANAS NA												
Partial mediation	.035 [.020, .047]	.053	.979	.975	323.62	260	.004				7537.01	7549.17
Full mediation	.035 [.021, .047]	.054	.978	.974	327.57	261	.003	5.29	1	.021	7538.62	7550.65
No TE indirect effect	.038 [.024, .049]	.055	.975	.970	336.57	261	.001	14.84	1	<.001	7548.06	7560.09
No TM indirect effect	.036 [.022, .048]	.054	.977	.973	330.31	261	.002	6.54	1	.011	7541.88	7553.91
sCORT												
Partial mediation	.036 [.021, .048]	.053	.978	.973	328.00	260	.003				7557.48	7569.65
Full mediation	.036 [.021, .047]	.054	.978	.974	328.50	261	.003	0.53	1	.469	7556.03	7568.06
No TE indirect effect	.036 [.021, .048]	.054	.978	.974	329.16	261	.003	1.17	1	.280	7556.77	7568.80
No TM indirect effect	.035 [.021, .047]	.053	.978	.974	328.11	261	.003	0.07	1	.785	7555.56	7567.58
sAA												
Partial mediation	.035 [.019, .047]	.053	.979	.975	324.60	260	.004				7280.28	7292.45
Full mediation	.034 [.019, .046]	.053	.980	.976	324.58	261	.004	0.56	1	.454	7279.34	7291.37
No TE indirect effect	.035 [.019, .047]	.053	.979	.975	327.02	261	.003	2.27	1	.132	7280.93	7292.96
No TM indirect effect	.035 [.021, .047]	.054	.978	.974	328.77	261	.003	4.68	1	.030	7282.33	7294.36

Note. CM = cognitive motivation; PANAS NA = Positive And Negative Affect Scale subscale for negative affect; VAS = visual analogue scale stress item; sCORT = salivary cortisol; sAA = salivary alpha-amylase; TE = task enjoyment; TM = task motivation; Δχ^2^ = χ^2^ difference in scaled χ^2^ difference test; Δ*df* = *df* difference between tested models; Δ*p* = *p*-value for scaled χ^2^ difference test of tested models; robust CFI, TLI, and RMSEA based on robust maximum likelihood estimator (MLR); sample size-adjusted BIC reported.

The effect of TM on the PANAS NA AUCi was β = .24 (*p* = .010) and the indirect effect via TM was β = .04 (*p* = .153). For TE the effect on stress was β = −.36 (*p* < .001), and the indirect effect was β = −.12 (*p* = .017). In this model, CM was directly associated with the AUCi (β = .15, *p* = .045). The indirect effect via TE but not that via TM was hence significant in both the model on the VAS item and PANAS NA and H4a is thus supported. Despite showing a trend in the hypothesized direction, H5a is rejected.

The sCORT AUCi was neither significantly related to TM (β = −.03, *p* = .784) nor TE (β = −.11, *p* = .290) or CM (β = .06, *p* = .483). The indirect effect was β = .00 (*p* = .795) with TM and β = −.04 (*p* = .328) with TE as mediator. The direct effect of TM (β = .19, *p* = .103) and TE (β = −.17, *p* = .239) on the sAA AUCi as well as the indirect effect via TM (β = .03, *p* = .211) and TE (β = −.06, *p* = .231) did not reach significance and the sAA AUCi (β = .09, *p* = .518) was also not significantly associated with CM. Neither the indirect effects via TE nor TM were significant in any of the models including physiological stress markers. Both H4b and H5b are hence rejected. Results of power analyses are included in the supplementary materials (Table S9).

## 4 Discussion

### 4.1 CM and stress

Among factors influencing stress during demanding intellectual problems, high CM may lead to greater familiarity with such challenges, a more skillful approach to coping as well as less mental discomfort and reduced frustration [3]. However, we did not find the hypothesized negative effect of CM on subjective stress during challenging reasoning tasks (H1a). Although physiological and subjective stress is often only weakly related [70], a weaker physiological response (H1b) might have been expected due to better adaptation and more constructive appraisals in high-CM individuals. One explanation for these null effects might be that purely cognitive challenges represent only moderately intense stressors (Fig. 3). Potential benefits of prior experience and more constructive appraisal in high-CM individuals may therefore not be sufficient to provoke such changes in reactivity. In sum, H1a and H1b are rejected.

### 4.2 TM and TE mediating the effect of CM on stress

A further explanation for the small effects of CM on stress may be multiple mediators with opposing effects on stress. According to Trait Activation Theory (TAT) [42], personality traits represent latent potentials for trait-specific behavior, cognitions, or emotions, given relevant cues. When high-CM individuals face intellectual problems, they should hence experience higher state motivation (H2) and enjoyment (H3).

Both hypotheses were supported, with a larger effect of CM on TE. NFC and interest-related EC are mainly approach-oriented traits, reflecting a disposition to seek out cognitively stimulating situations. Within the Intellect framework, they align more with seeking than conquering [7], consistent with findings on exploratory behavior [5] and need for closure [71]. Yet TM items were administered right after completion and were hence likely also answered with regard to motivation for sustained effort and willingness to keep working rather than just initial approach motivation. In contrast, the CM-specific enjoyment of cognitive challenges tends to manifest mainly *during* intellectual activity and may have therefore been better captured by TE items.

Furthermore, according to Self-Determination Theory (SDT) [32] autonomy plays a key role in intrinsic motivation and self-selection of goals and challenges may thus be central to motivational engagement. Since participants could neither choose the tasks nor whether to engage with them, motivational engagement may have been reduced. However, TM in the present study may in fact not only reflect *intrinsic* motivation. The results are also consistent with findings suggesting higher motivation to avoid failure in high-NFC individuals when expecting difficult tasks, possibly reflecting a desire to avoid self-perceptions of incompetence [72]. Moreover, it is not clear how intrinsic motivation to approach intellectual problems related to high CM could explain the significant positive effects of TM on the VAS stress item and PANAS NA AUCi. Plausibly, the variance in TM shared with stress markers may rather reflect higher personal relevance of the performance on the tasks [73]. While this cannot be determined for the effect of CM on TM, the effect of TM on the stress AUCi is possibly attributable to TM variance reflecting extrinsic motivation, such as motivation to maintain a positive self-image.

As observed previously [44], we found TE to be negatively related to stress, the effect being significant for the subjective markers, but not for sCORT or sAA. Since CM was associated with both TM and TE, this yields an interesting pattern for subjective stress: While the situational manifestation of CM as TE suggests a stress-buffering effect, TM is positively associated with it. However, the only significant indirect effects included TE as mediator (H4) and subjective stress as outcome, the indirect effect via TM (H5) only showing a trend. Moreover, these patterns are limited to subjective stress markers and did not generalize to physiological stress. Overall, the relationship of CM with cognitive stress may be more complex than expected. But since the pattern is inconsistent across markers, findings should be interpreted cautiously.

Differences in indirect effects on subjective versus physiological stress are attributable to the generally weaker associations of TM and TE with physiological measures. One reason may be that state motivation and affect should be more closely aligned with subjective appraisals of stress [20], as all are fundamentally psychological constructs. Subjective stress reflects how demands are interpreted relative to coping resources, a process which might be more directly influenced by motivational and affective states than autonomic or neuroendocrine responses.

Furthermore, according to the Biopsychosocial Model of Challenge and Threat (BPS) [24], specific physiological response patterns reflect whether a situation is appraised as a threat or a challenge. While the threat pattern is characterized by *both* SAM and HPA activity, *exclusive* SAM activation reflects challenge responses [24]. Effects involving sAA as a SAM marker were somewhat lower than those for subjective stress. However, the slight sAA increases around the stressor and lacking increase in sCORT levels suggests that the task was experienced as a challenge rather than a threat. TM may capture the perceived importance of success, increasing engagement and potentially stress, yet the lack of a threat appraisal may have prevented increased sCORT responses in high-TM participants.

Moreover, the effort invested in a task can be conceptualized as the mobilization of resources, which increases proportionally with task demands given a possibility of success [[74], [75], [76]]. In contexts in which performance can vary as a function of participants’ actions, stronger activation of the sympathetic nervous system (SNS) has been suggested to represent a physiological indicator of such a resource mobilization. In the present study, situationally manifested task motivation is likely to increase the maximally justifiable effort to expend on the administered tasks [76]. Yet, since high-CM individuals are particularly drawn to complex cognitive tasks, those administered in the present study may also have provoked increased effort expenditure [see e.g., 13,43]. Given the above characterization, this suggests higher effort and thus potentially higher sAA levels as reflections of SNS activity [76]. Although the small to moderate effect of TM on sAA did not quite reach significance, its direction and size is consistent with these considerations. High-CM individuals, however, should not only invest more effort in challenging tasks. As suggested above, they may also possess means for adaptive coping and regulation of potentially arising stress. Reflected in a remaining negligible positive association, the potentially resulting opposing effects of CM on the sAA AUCi could thus, to some extent, have cancelled each other out.

### 4.3 Limitations

Note that several possible explanations discussed above should be considered tentative, since factors such as task familiarity and coping strategies were not directly measured. Methodological limitations should hence be considered additionally. Overall, stress responses around stressor onset were modest compared to paradigms involving psychosocial or physical stress (e.g., the TSST [77]). As suggested also by Fig. S1 in the supplementary materials, this may have resulted in floor effects in stress responses, particularly in case of sCORT. The lack of effects of CM on the AUCi might consequently also be due to reduced variance in stress outcomes.

Furthermore, while sAA reflects SNS activation [78], it may not be able to cleanly distinguish SNS from parasympathetic nervous system (PNS) activation [e.g., 79]. The lack of an association of CM with the sAA AUCi might hence partly be due to the latter not being specific enough for stress-related changes in sympathetic activity. This limitation could have been addressed by including more specific SNS indicators such as the pre-ejection period or the skin conductance response alongside PNS indicators like the heart rate variability [76,80,81]. In addition, unlike other ANS indicators, the limited number of saliva samples only allowed for a coarse temporal resolution of physiological activation, potentially missing more transient changes in response to the tasks. Also relating to the stress markers, the timing of the sample collection was based on the goal of capturing physiological stress simultaneously with the subjective experience. Since the total number of physiological samples was limited, the timing of saliva collection was optimized for the assessment of sCORT and thus reflected the latency of the HPA-axis response relative to stressor onset. However, as the tasks may have represented a challenge rather than a threat, a stronger focus on sAA and further ANS indicators could have yielded additional insights.

Several constraints in the project did not allow for a greater sample size. Results of post-hoc power analyses suggest sufficient or higher power in analyses relying on conventionally considered RMSEA thresholds. Using cut-off values based on dynamic goodness of fit index (GOF) simulations, power to reject a close fit when misfit is in fact strong was excellent. However, it was occasionally lower when misfit was only moderate, suggesting a cautious evaluation of model fit.

A final limitation concerns the temporal ordering assumed in mediation models. Stress was measured over a longer period before, during and after the tasks. TE and TM were measured afterwards, although explicitly referring to states during the tasks. Conceptually, TM arguably reflects the subjective relevance of a task, such as performing well, avoiding perceptions of incompetence etc., which may trigger stress. The reverse, i.e., that stress increases the relevance of the task, seems conceptually far less plausible. Similarly, TE is likely to precede stress level changes given its known buffering effect [44] as well as faster recovery in individuals experiencing more positive mood [82]. Finally, work on the task began before the third measurement and hence prior to most measurements that were the basis of the AUCi. Overall, although temporal precedence cannot be conclusively clarified, this supports the assumption that TE and TM influence stress rather than the reverse.

## 5 Conclusion

The present study examines the interplay of cognitive motivation (CM) and cognitive stress, focusing on the mediating roles of task motivation (TM) and task enjoyment (TE). While CM was not directly associated with subjective or physiological stress, our results highlight the importance of considering indirect pathways. CM was positively related to both TM and TE, which showed significant but opposing associations with subjective stress: Higher motivation may in fact lead to increased stress and enjoyment to a reduction. Although this is consistent with a full mediation model, indirect effects were significant only in some cases. Moreover, this pattern did not extend consistently to physiological stress markers as only sAA was similarly related to TE and TM. Our findings highlight the complexity of motivational and affective mechanisms underlying cognitive stress and provide first evidence for a double-edged role of CM in such contexts. Future research may build on these experimental findings by examining them in real-world settings using more common stressors.

## Author note

We have no conflict of interest and no funding sources to disclose. The study was preregistered at OSF Registries: https://osf.io/yv2e3/overview.Associated materials can be accessed at: https://osf.io/v7qdn/overview (files are attached as supplementary materials and will be uploaded upon acceptance). Early results of this study have been presented at the 52. DGPs Congress 2022 in Hildesheim and at the International Society for the Study of Individual Differences (ISSID) Conference 2025 in Vienna. Correspondence should be addressed to Felix M. Schweitzer, MSB Medical School Berlin, Rüdesheimer Str. 50, 14197 Berlin, Germany.

## Funding

This research did not receive any specific grant from funding agencies in the public, commercial, or not-for-profit sectors.

## CRediT authorship contribution statement

**Felix M. Schweitzer:** Conceptualization, Data curation, Formal analysis, Investigation, Methodology, Project administration, Resources, Software, Visualization, Writing – original draft, Writing – review & editing. **Sören Enge:** Conceptualization, Investigation, Methodology, Project administration, Resources, Supervision, Validation, Writing – review & editing. **Julian Hellmann-Regen:** Formal analysis, Investigation, Resources, Validation. **Monika Fleischhauer:** Conceptualization, Investigation, Methodology, Project administration, Resources, Supervision, Validation, Writing – review & editing.

## Declaration of competing interest

The authors declare that they have no known competing financial interests or personal relationships that could have appeared to influence the work reported in this paper.
