# Supplementary material for: The two faces of cognitive motivation in cognitive stress: The stress-reducing effect of positive affect and the stress-intensifying effect of motivation
Source: Compr Psychoneuroendocrinol. 2026 Jul 10;27:100362. doi: 10.1016/j.cpnec.2026.100362 (PMC13377448; doi:10.1016/j.cpnec.2026.100362)
Supplement: Multimedia component 1 [file mmc1.docx]

**1. Data preparation**

**1.1 Transformation of stress data**

Since stress marker distributions were strongly right-skewed, Box-Cox transformations (Saskia, 1992) were applied. As suggested by Miller and Plessow (2013), for different values of λ (ranging from 0 to 1), the χ^2^ -statistics from the Doornik-Hansen (DH) (Doornik & Hansen, 2008) and Breusch-Pagan (BP) (Breusch & Pagan, 1979) were obtained to quantify violations of normality and homoskedasticity assumptions, respectively. BP and DH test χ^2^ values, as a function of different values of λ, were plotted and the optimal λ for the transformation of each stress marker was defined as the value corresponding to the lowest mean χ^2^.

**1.2 Careless responding**

We screened survey data for careless responding (Leiner, 2019; Ward & Meade, 2023) using several established indicators such as response variance, longest string of identical responses, Mahalanobis distance, and average item response time. Additionally, we assessed intra-person correlations between empirically and conceptually related item pairs (synonyms/antonyms), following the approach by Ward and Meade (2023). Item pairs with correlations > .60 and < -.40 were treated as synonyms and antonyms, respectively, and used to calculate intra-person correlations between these two sets of pairs. Since it is difficult to arrive at clear cut-off values, we screened for unusual values on multiple indicators (e.g., long strings, low variance, low synonym correlation). Cut-offs for flagging participants were an average item response time < six seconds, a total sum across scale longstrings > 45, Mahalanobis distance flags on more than two scales, synonym intra-person correlations < .60 or antonym correlations > -.40, and item response variation < 2 SD below the mean on at least one scale. To preserve as much as possible of the comparatively small sample size for the SEMs, only clearly identifiable cases were excluded. These were defined as participants flagged on more than three of the mentioned criteria.

**1.3 Factor structure and item parceling**

Due to the relatively small sample size, latent constructs were modelled using item parceling (Little et al., 2002; Little et al., 2013). NFC, intellect, and EC scale factor structures were first investigated with confirmatory factor analyses (CFAs) and items were subsequently assigned to parcels based on factor loadings. Model fits for item-level and parcel-based measurement models are displayed in Table 1.

To model NFC, a CFA item-level model with one content factor and two orthogonal method factors for positively and negatively phrased items was estimated (Zhang et al., 2018) and showed substantially better fit than the simple single content factor model. Items were then assigned to parcels such that the item with the highest loading on the content factor was paired with the item with the lowest loading in the first parcel, the second highest with the second lowest in the second parcel, and so forth. This was repeated with the remaining items until all were allocated (Little et al., 2002), resulting in four parcels (four items each). Mean/sum item loadings across parcels ranged from .50 to .54 / 2.01 to 2.17.

The multidimensional intellect model (Mussel, 2013) showed better fit than a single factor model and models specifying only subsets of factors (*seek*/*conquer* and *think*/*learn*/*create*). This model was used to assign items to parcels with the goal of equally representing *operations* and *processes* in terms of loadings. Mean/sum loadings across parcels ranged from .30 to .35 / 0.90 to 1.04 on the *seek* factor, from .40 to .50 / 1.19 to 1.50 on the *conquer* factor, from .57 to .70 / 1.14 to 1.39 on the *learn* factor, .54 to .71 / 1.08 to 1.42 on the *create* factor, and .63 to .68 / 1.26 to 1.36 on the *think* factor.

There appears to be no prior confirmatory assessment of the *diverse*/*specific* subscales of the German EC scale (Renner, 2006). The best fitting model was a bifactor model with a *diverse*-, a *specific*-, and a general EC factor (correlations with the other two constrained to 0), fitting better than a single factor model, a model including only *diverse* and *specific* factors, and a second-order factor model. Based on the bifactor model, we assigned items so that the loadings on the general EC factor were distributed as evenly as possible across parcels. This resulted in two parcels comprising two items of each of the subscales and one of the respective other. The third parcel was assigned two items of each one. Mean/sum loadings ranged from .54 to .61 / 1.77 to 2.16.

**1.4 Software**

Statistical analyses were done using R Studio, version 2024.4.2.764 (Posit Team, 2024) and main functions were *sem*, *cfa*, and *anova* of the *lavaan* package, version 0.6.19 (Rosseel, 2012), *reliability* (*semTools* package, version 0.5-7*,* Jorgensen et al., 2025)*, semPower* (*semPower* package, version 2.1.3, Moshagen & Bader, 2025), *irv*, *longstring*, and *mahad* (*careless* package, version 1.2.2, Yentes & Wilhelm, 2023).

**2. Type I error risk correction in multiple testing**

Considerations by Rubin (2017, 2021), with antecedents in Turkheimer et al. (2004) and Matsunaga (2007), provide a rationale for Type I error risk correction with a considerably more precise and justified answer to the question than the rigid views occasionally held in favor of one extreme or another (e.g., correcting for *all* tests in a study or none). In the present study, we hence applied these conclusions to our analyses and corrected for Type I error risk based on the number of tests conducted to answer a specific hypothesis.

**2.1 Structural equation models**

We initially intended to model subjective and physiological stress as a latent factor based on two indicators each. This was abandoned since the physiological stress markers shared very little variance. Because we thus examined the respective markers in distinct tests, these tests are theoretically linked by testing the same hypothesis. For example, this was the case in the hypothesis of cognitive motivation (CM) negatively predicting physiological stress as two significance tests were conducted to test it: one including salivary alpha-amylase (sAA) and one including salivary cortisol (sCORT) as stress marker. In such cases, the two associated tests were hence considered a family. Hypotheses were tested conjunctively, and a null hypothesis was thus rejected only if the effect was significant in both tests (Rubin, 2021).

**2.2 Comparison of pre-stressor and stressor stress measurements**

We also tested whether the stress levels of the individual markers increased significantly from pre-stressor to stressor measurement times. This was addressed using two comparisons (between T0 and T2 and between T1 and T2) in case of PANAS NA, the VAS stress item, and sCORT and one (T0 and T1) in case of sAA. Except for the latter, these tests hence constitute a family of two per stress marker. However, the pre-stressor period and the stressor period mark very specific phases in the experiment. As we were interested in whether there is the expected clearly identifiable increase during the stressor and hence both in comparison to a measurement shortly before and the earlier baseline measurement, conjunctive testing was applied. Therefore, it was only concluded that there is indeed a difference between pre-stressor and stressor stress levels if all associated tests turned out significant.

**3. Descriptive statistics and correlation analyses**


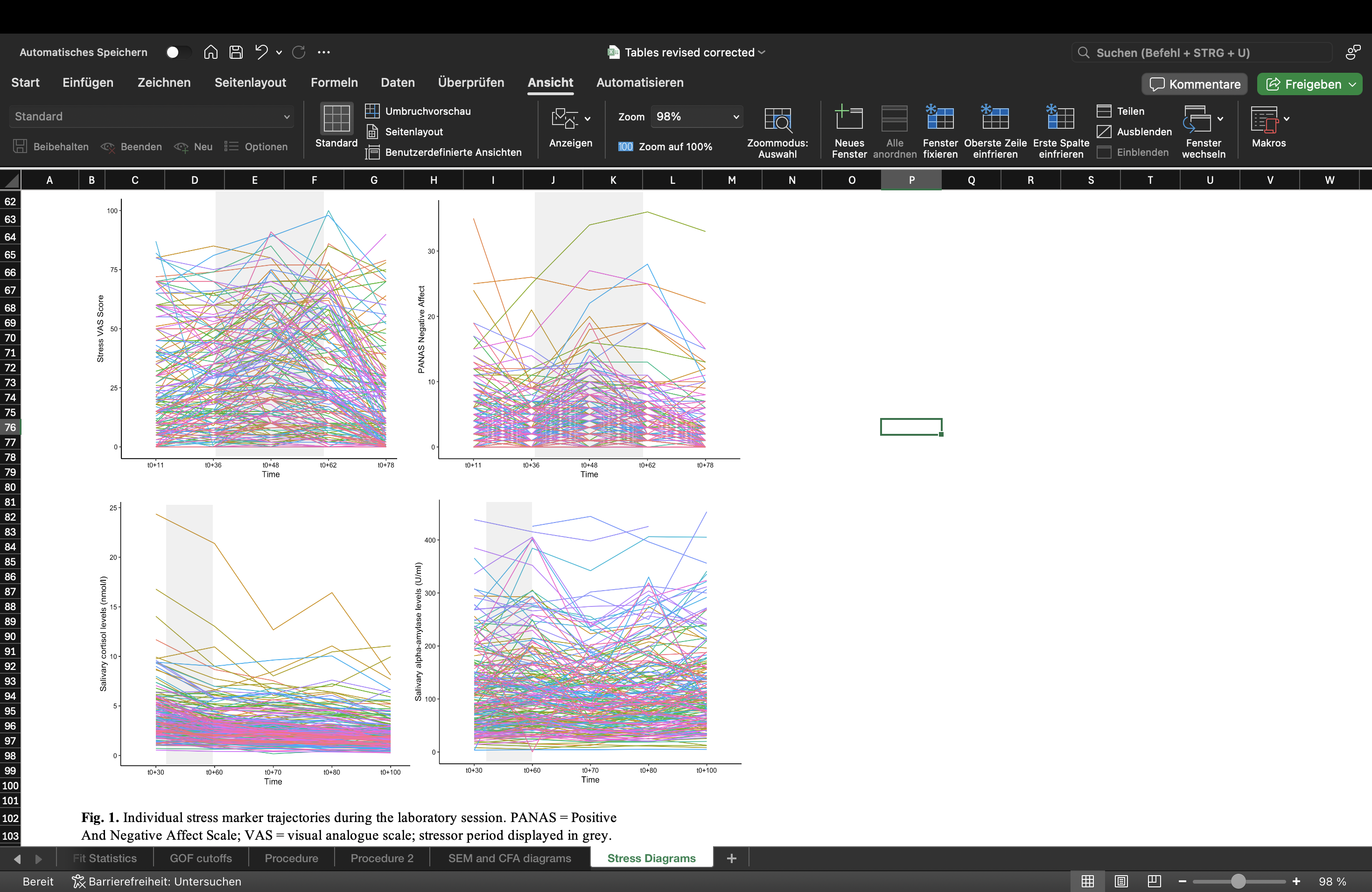
**4. Stress marker data**


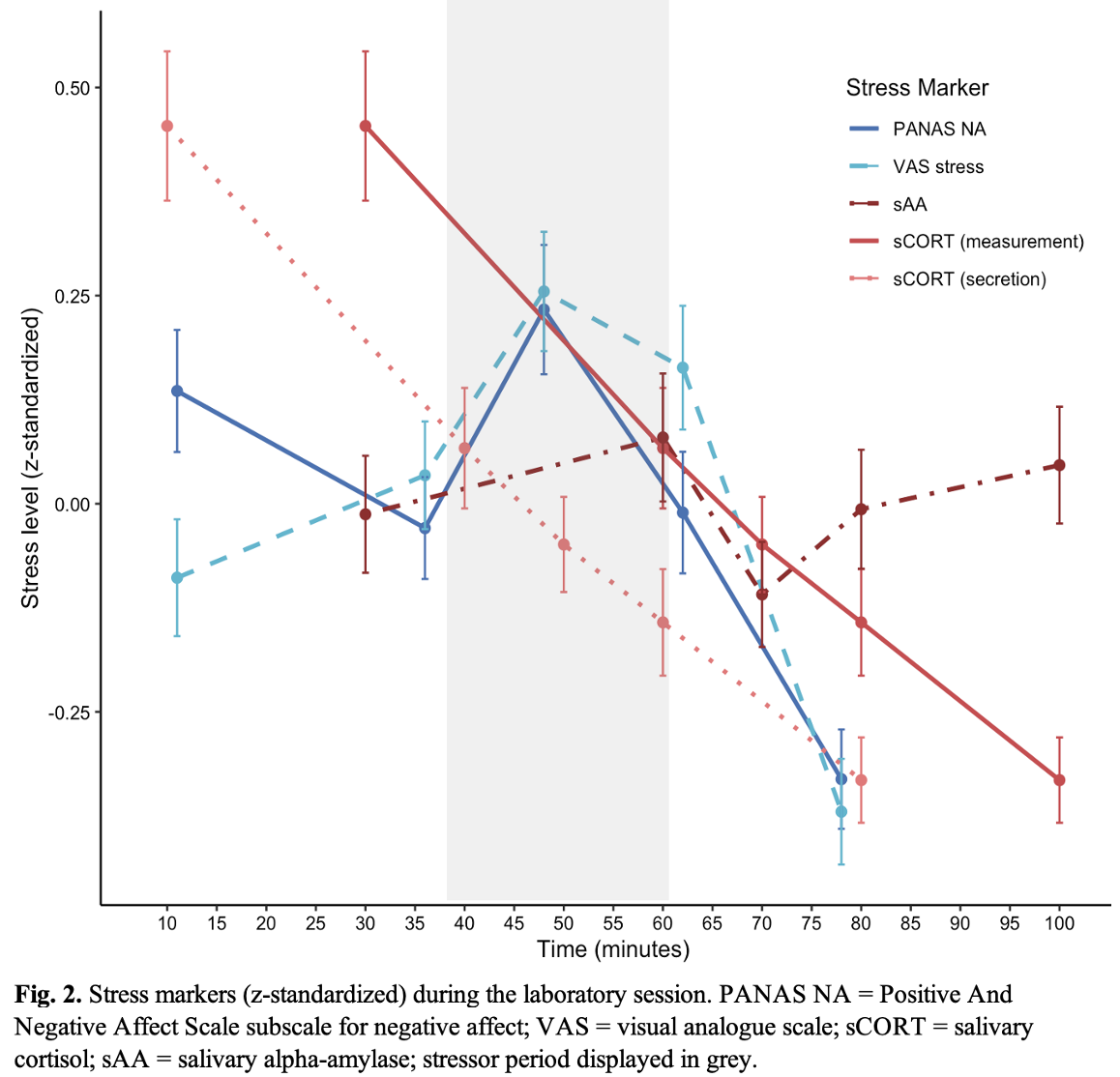


**5. Significance tests of stress-level changes**

One-sided paired Wilcoxon tests were conducted to test whether participants responded to the stressor. The increase in PANAS NA levels from T0 (*M* = 4.58, *SD* = 5.05) to T2 (*M* = 5.04, *SD* = 5.35) was significant (*r* = .13, *p* = .045), as was that between T1 (*M* = 3.74, *SD* = 4.19) and T2 (*r* = .39, *p* < .001). The increase in the VAS stress item was significant (*r* = .54, *p* < .001) between T0 (*M* = 22.84, *SD* = 22.26) and T2 (*M* = 30.36, *SD* = 22.70) and similarly (*r* = .49, *p* < .001) between T1 (*M* = 25.52, *SD* = 20.50) and T2. sCORT stress levels were continuously decreasing, and differences were hence not tested for significance. As sAA and sCORT were collected together and sAA concentration reflects the stress response more immediately (Smeets et al., 2012), only T0 (*M* = 114.24, *SD* = 81.14) and T1 (*M* = 121.58, *SD* = 88.54) can be interpreted as measurement times in vicinity of the stressor. The effect size for the difference here was *r* = .12 (*p* = .042). Only in case of the VAS stress item and sAA was there hence a consistent increase from pre-stressor to stressor levels across all tests.

**6. Model-specific goodness-of-fit indices**

Recent evidence on cutoff values for goodness-of-fit indices (GOFs) in CFA and SEM suggests that these can depend on features beyond just the degree of model misfit, such as size of residual variances, sample size, and type of estimator (e.g., Heene et al., 2011; DiStefano et al., 2019; Xia & Yang, 2019; Groskurth et al., 2024). Usually applied cutoff values (e.g., Hu & Bentler, 1999) may hence not be applied to substantially different models. However, a solution to this problem is model-specific simulations with the goal of estimating cutoffs for the model under investigation (e.g., Groskurth et al., 2024). Next to the traditionally applied GOFs, we thus conducted simulations to obtain such cutoffs.

For each of the models with a different stress marker AUCi as outcome, we simulated 4000 data sets. As suggested by the analyses in the main text, the models in which the effect of CM on the stress outcome was constrained to zero were not significantly worse fitting than the models not constraining this path. More importantly, using this model as the analysis model allows for using the path from CM to stress to induce different degrees of misfit that is comparable in size across stress outcomes (e.g., effects of β = -.10, β = -.20, and β = -.30 in the population, not reflected in the model). We hence chose this model as a common basis. 1000 datasets were sampled based on coefficients, variances and covariances obtained from this specification. For the remaining 3000 datasets, we simulated data based on models that differed to varying degrees from this model. The first 1000 of these represented a mild divergence, the second 1000 a moderate divergence, and the third 1000 a strong divergence. A summary of this is displayed in Table 7.

The analysis model was fitted to each of these four datasets and GOFs were recorded. Relative to the simulated data, this model was hence either perfectly fitting (“true model”) or mildly, moderately, and strongly misspecified. This allows for obtaining three cutoff values by determining the respective GOF value at which at least 95% of misspecified models are correctly and no more than 5% of correctly specified models are incorrectly rejected (McNeish & Wolf, 2023). As suggested by McNeish and Wolf (2023), this threshold was relaxed to 90% each if it was not possible to obtain distributions for which a cutoff with the former properties could be derived. If no value could be determined that met at least the relaxed requirements even with this large number of simulations, no cutoff was derived. As displayed in Table 8, this was only so for the SRMR in most of the mild and moderate conditions.

**7. Power analyses**

Finally, we evaluated the power to detect meaningful levels of misfit, following the procedure proposed by MacCallum et al. (1996). Here, power is calculated using two RMSEA values – one representing the null hypothesis and one representing the alternative hypothesis. MacCallum et al. (1996) suggest the RMSEA-based power analysis as a test of close fit, in which the null hypothesis reflects a fit (RMSEA0) that is less than or equal to a chosen close-fit threshold (e.g., 0.05). Power is then evaluated by choosing an alternative RMSEA value (RMSEAa) that reflects a relevant level of misfit (e.g., 0.08) and estimating the probability of rejecting the close-fit hypothesis when the actual level of fit is represented by the higher RMSEA. It is thus understood as the probability of detecting and rejecting the hypothesis of a close fit when the model in fact has a poor fit in the population.

The first cluster of analyses was based on these conventional cut-off values, which were used together with other model-specific parameters (*N*, *df*, and α) to obtain power. However, the above-reported simulations for model-specific GOF cut-offs are specifically aimed at deriving meaningful model-specific values to determine if a given model fits the data (Groskurth et al., 2024). We hence also conducted analyses based on these RMSEA values additional to those relying on the mentioned conventional ones. Specifically, for each model we used the simulation-based RMSEA close-fit cutoff as the RMSEA0, with values below this cut-off indicating close fit. To allow for a more nuanced evaluation, we then conducted two power analyses per model: one using the simulation-derived moderate misfit RMSEA as RMSEAa, and another using the corresponding strong misfit RMSEA as RMSEAa. The analyses were conducted using the *findRMSEApower* function (*semPower* package, version 2.1.3, Moshagen & Bader, 2025). Results are reported in Table 9. Given the smaller difference between fit thresholds, they suggest lower power to reject the hypothesis of a close fit when based on the moderate misfit cut-offs from the GOF simulations as RMSEAa. However, power is excellent when using the conventionally chosen RMSEA values (i.e., 0.05 and 0.08) as well as the strong misfit cut-offs from the GOF simulations as RMSEAa.

**References**

Breusch, T. S., & Pagan, A. R. (1979). A simple test for heteroscedasticity and random coefficient variation. *Econometrica: Journal of the Econometric Society*, 1287–1294. <https://doi.org/10.2307/1911963>.

DiStefano, C., McDaniel, H. L., Zhang, L., Shi, D., & Jiang, Z. (2019). Fitting large factor analysis models with ordinal data. *Educational and Psychological Measurement, 79*(3), 417–436. <https://doi.org/10.1177/0013164418818242>.

Doornik, J. A., & Hansen, H. (2008). An omnibus test for univariate and multivariate normality. *Oxford Bulletin of Economics and Statistics*, *70*, 927–939. <https://doi.org/10.1111/j.1468-0084.2008.00537.x>.

Groskurth, K., Bluemke, M., & Lechner, C. M. (2024). Why we need to abandon fixed cutoffs for goodness-of-fit indices: An extensive simulation and possible solutions. *Behavior Research Methods*, *56*(4), 3891-3914. <https://doi.org/10.3758/s13428-023-02193-3>.

Heene, M., Hilbert, S., Draxler, C., Ziegler, M., & Bühner, M. (2011). Masking misfit in confirmatory factor analysis by increasing unique variances: a cautionary note on the usefulness of cutoff values of fit indices. *Psychological Methods*, *16*(3), 319–336. [https://doi.org/10.1037/a0024917](https://psycnet.apa.org/doi/10.1037/a0024917).

Hu, L.-T., & Bentler, P. M. (1999). Cutoff criteria for fit indices in covariance structure model: Conventional criteria versus new alternatives. *Structural Equation Modeling, 6*(1), 1–55. <https://doi.org/10.1080/10705519909540118>.

Jorgensen, T. D., Pornprasertmanit, S., Schoemann, A. M., Rosseel, Y., Miller, P., Quick, C., Garnier-Villarreal, M., Selig, J., Boulton, A., Preacher, K., Coffman, D., Rhemtulla, M., Robitzsch, A., Enders, C., Arslan, R., Clinton, B., Panko, P., Merkle, E., Chesnut, S., Byrnes, J., Rights, J. D., Longo, Y., Mansolf, M., Ben-Shachar, M. S., Rönkkö, M., Johnson, A. R. , Vanbrabant, L. (2025). *Package ‘semtools’* (Version 0.5-7) [R package semTools]. <https://cran.r-project.org/web/packages/semTools/semTools.pdf>.

Leiner, D. J. (2019). Too fast, too straight, too weird: Non-reactive indicators for meaningless data in internet surveys. *Survey Research Methods*, *13*(3), 229–248. <https://doi.org/10.18148/srm/2019.v13i3.7403>.

Little, T. D., Cunningham, W. A., Shahar, G., & Widaman, K. F. (2002). To parcel or not to parcel: Exploring the question, weighing the merits. *Structural Equation Modeling, 9*(2), 151–173. <https://doi.org/10.1207/S15328007SEM0902_1>.

Little, T. D., Rhemtulla, M., Gibson, K., & Schoemann, A. M. (2013). Why the items versus parcels controversy needn’t be one. *Psychological Methods*, *18*(3), 285–300. [https://doi.org/10.1037/a0033266](https://psycnet.apa.org/doi/10.1037/a0033266).

MacCallum, R. C., Browne, M. W., & Sugawara, H. M. (1996). Power analysis and determination of sample size for covariance structure modeling. *Psychological Methods*, *1*(2), 130–149.

Matsunaga, M. (2007). Familywise error in multiple comparisons: Disentangling a knot through a critique of O’Keefe’s arguments against alpha adjustment. *Communication Methods and Measures, 1,* 243–265. <https://doi.org/10.1080/19312450701641409>.

McNeish, D., & Wolf, M. G. (2023). Dynamic fit index cutoffs for one-factor models. *Behavior Research Methods, 55*(3), 1157-1174. <https://doi.org/10.3758/s13428-022-01847-y>.

Miller, R., & Plessow, F. (2013). Transformation techniques for cross-sectional and longitudinal endocrine data: Application to salivary cortisol concentrations. *Psychoneuroendocrinology, 38*(6), 941–946. <https://doi.org/10.1016/j.psyneuen.2012.09.013>.

Moshagen, M., & Bader, M. (2025). *Package ‘semPower’* (Version 2.1.3) [R Package semPower]. <https://cran.r-project.org/web/packages/semPower/index.html>.

Mussel, P. (2013). Intellect: a theoretical framework for personality traits related to intellectual achievements. *Journal of Personality and Social Psychology*, *104*(5), 885–906. [https://doi.org/10.1037/a0031918](https://psycnet.apa.org/doi/10.1037/a0031918).

Posit Team (2024). *RStudio: Integrated development environment for R* (Version 2024.4.2.764) [Computer software]. Posit Software. <http://www.posit.co/>.

Renner, B. (2006). Curiosity about people: The development of a social curiosity measure in adults. *Journal of Personality Assessment, 87*(3), 305–316. <https://doi.org/10.1207/s15327752jpa8703_11>.

Rosseel, Y. (2012). lavaan: An R package for structural equation modeling. *Journal of Statistical Software, 48*(2), 1–36. <https://doi.org/10.18637/jss.v048.i02>.

Rubin, M. (2017). Do p values lose their meaning in exploratory analyses? It depends how you define the familywise error rate. *Review of General Psychology, 21*(3), 269–275. <https://doi.org/10.1037/gpr0000123>.

Rubin, M. (2021). When to adjust alpha during multiple testing: A consideration of disjunction, conjunction, and individual testing. *Synthese, 199*(3), 10969–11000. <https://doi.org/10.23668/psycharchives.4960>.

Saskia, R. M. (1992). The Box-Cox transformation technique: a review. *Journal of the Royal Statistical Society Series D: The Statistician*, *41*(2), 169–178. <https://doi.org/10.2307/2348250>.

Smeets, T., Cornelisse, S., Quaedflieg, C. W., Meyer, T., Jelicic, M., & Merckelbach, H. (2012). Introducing the Maastricht Acute Stress Test (MAST): a quick and non-invasive approach to elicit robust autonomic and glucocorticoid stress responses. *Psychoneuroendocrinology, 37*(12), 1998–2008. <https://doi.org/10.1016/j.psyneuen.2012.04.012>.

Turkheimer, F. E., Aston, J. A., & Cunningham, V. J. (2004). On the logic of hypothesis testing in functional imaging. *European Journal of Nuclear Medicine and Molecular Imaging, 31*, 725–732. <https://doi.org/10.1007/s00259-003-1387-7>.

Ward, M. K., & Meade, A. W. (2023). Dealing with careless responding in survey data: Prevention, identification, and recommended best practices. *Annual Review of Psychology*, *74*(1), 577–596. <https://doi.org/10.1146/annurev-psych-040422-045007>.

Xia, Y., & Yang, Y. (2019). RMSEA, CFI, and TLI in structural equation modeling with ordered categorical data: The story they tell depends on the estimation methods. *Behavior Research Methods*, *51*, 409-428. <https://doi.org/10.3758/s13428-018-1055-2>.

Yentes, R., & Wilhelm, F. (2023). *Package ‘careless’* (Version 1.2.2) [R Package careless]. <https://cran.r-project.org/web/packages/careless/index.html>.

Zhang, Y., Klopp, E., Dietrich, H., Brünken, R., Krause, U. M., Spinath, B., Stark, R., & Spinath, F. M. (2018). Reexamining the factorial validity of the 16-item scale measuring Need for Cognition. *European Journal of Psychological Assessment, 36*(1), 212–215. <https://doi.org/10.1027/1015-5759/a000484>.
